# Myopic Progression in Girls with Gonadotrophin-Releasing Hormone Agonist Treatment for Central Precocious Puberty

**DOI:** 10.3390/children8030171

**Published:** 2021-02-24

**Authors:** Seung Ah Chung, Hae Sang Lee, Seung Woo Kim, Jin Soon Hwang

**Affiliations:** 1 Department of Ophthalmology, Ajou University School of Medicine, Suwon 16499, Korea; mingming8@naver.com (S.A.C.); 108557@aumc.ac.kr (S.W.K.); 2 Department of Pediatrics, Ajou University School of Medicine, Suwon 16499, Korea; pedhwang@ajou.ac.kr

**Keywords:** myopia, progression, precocious puberty, axial length

## Abstract

We sought to determine whether the myopic progression of patients with central precocious puberty (CPP) who were undergoing treatment differed from that of their healthy peers with normal pubertal onset and progression. Eighteen girls with CPP and 14 age-matched controls who underwent regular ophthalmic examinations for at least 1 year were included. All the CPP patients received a 3.75 mg leuprolide acetate depot subcutaneously every 28 days. The spherical equivalent (SE) and axial length (AL) for myopia progression and the pubertal parameters (height, body weight, body mass index, Tanner stage, and bone age) were compared between the two groups. Of 32 subjects with a mean age of 8.6 ± 0.7 years, the SEs and ALs did not differ at baseline between the two groups, which had similar weight and similar body mass index. After 1 year, both the CPP patients and controls showed myopic progression, with an average myopic shift of −0.73 ± 0.48 diopters (D) and AL elongation with a mean change of 0.44 ± 0.61 mm. The SE and AL changes over 1 year were greater in the controls than those in the CPP patients, which was not statistically significant (–0.85 ± 0.55 D vs. –0.64 ± 0.41 D and 0.55 ± 0.89 mm vs. 0.35 ± 0.22 mm, respectively). The change in AL correlated significantly with the change in the height (*β* = 0.691, *p* = 0.039). In this 1-year study, the CPP patients with treatments trended to show less myopic progression than the controls.

## 1. Introduction

Myopia is the most common ocular disorder, with increasing prevalence in recent decades, predominantly in East Asia [1]. Myopia is a refractive condition that is usually attributed to excessive axial length (AL) elongation and mostly progresses during school ages, especially during growth spurts [2,3]. Using data from the Korean National Survey, Lyu et al. [4] reported that a younger age at menarche was associated with an increased risk of severe myopia. They suggested an increase in female sex hormones, such as estrogen, or growth spurts during puberty may be associated with the severity of myopia. Furthermore, the population-based Generation R cohort study in Copenhagen found an association between body height or pubertal maturation and subfoveal choroidal thickness, which is known as a biometric parameter of myopic progression [5]. These studies suggested that puberty may affect the progression of myopia. 

Central precocious puberty (CPP) is characterized by early hypothalamic-pituitary-gonadal axis activation. When girls and boys show signs of puberty and its progression before the ages of 8 and 9, respectively, it is considered precocious puberty [6]. Clinically, patients with CPP experience growth spurts, rises in pubertal hormones, and bone age advancements. Gonadotropin-releasing hormone agonists (GnRHas) are the treatment of choice for CPP and have been widely used for decades [7]. The primary aim of GnRHa treatment for patients with CPP is the effective and selective suppression of gonadal sex steroid secretion to stop premature sexual maturation. 

We postulate that pubertal hormone and growth spurt regulation may contribute to slower rates of myopic progression during GnRHa treatment. In this study, we aimed to evaluate myopic progression in the CPP patients who received GnRHa treatment as denoted by the AL and the spherical equivalent (SE), compared with that of age-matched controls with normal pubertal onset and progression.

## 2. Materials and Methods

### 2.1. Study Population

We reviewed the records of all the girls with CPP who were treated with GnRHas at Ajou University Hospital between 2010 and 2018. A total of 18 girls with CPP who had regular ophthalmic examinations for at least 1 year were included in this study. The inclusion criteria were as follows: (i) objective breast engorgement that appeared before the age of 8 years, (ii) an advanced bone age more than 1 year over the chronological age, and (iii) a peak luteinizing hormone (LH) level above 5 IU/L during a GnRH stimulation test. We excluded subjects who had precocious puberty with an identified etiology, such as a brain tumor or cranial irradiation. The patients’ plasma thyroxin and thyroid stimulating hormone concentrations were measured to exclude hypothyroidism. Patients with anisometropia of >1.00 diopters (D), astigmatism of >2.00 D, and hypermetropia of > 0.5 D were excluded. Patients with ocular diseases other than refractive errors, including amblyopia, and intermittent exotropia of >15 prism diopters; those who underwent previous interventions for myopia control such as atropine treatment or orthokeratology lens use; and those with systemic diseases other than CPP were also excluded. The age-matched control group consisted of 14 healthy girls who experienced normal pubertal onset and progression. The control subjects also had regular ophthalmic examinations, and their anthropometric measurements were monitored for at least 1 year. 

### 2.2. Ophthalmic Measurements for Myopic Progression

All subjects underwent a complete ophthalmic examination including cycloplegic refraction and AL measurement at baseline and 1-year follow-up. For the patients with CPP, the start of the GnRHa treatment and initial ophthalmic examination was within a 6-month interval (mean interval: 0.2 ± 0.4 years). Cycloplegic refraction was performed one hour after instillation of three cycles of eye drops. Two separate eye drops, cyclopentolate 1% (OcuCyclo, Samil, Korea) and tropicamide 1% (Mydrin-P, Santen, Japan), were administered to both eyes 5 min apart. A second cycle of the same cycloplegic drops were administered 10 min after the first cycle. A third cycle of the same cycloplegic eye drops was administered 30 min after the second cycle if the pupillary light reflex was still present. The cycloplegic SE was calculated as the spherical power plus half of the cylinder power. The ocular AL was measured using an IOL Master 500 (Carl Zeiss Meditec AG, Jena, Germany), based on noncontact partial coherence interferometry. Five readings, with a maximum-minimum deviation of 0.05 mm or less, were taken and averaged. Myopic progression was defined as a change in the SE and AL during the study period. The annual myopic progression rate was calculated as the change in the SE and AL over each year. 

### 2.3. Puberty Parameters Measurements

GnRH stimulation tests were performed to assess the pubertal status in 18 patients. Basal serum samples were evaluated prior to injecting GnRH (100 µg gonadorelin [Relefact^®^]; Sanofi-Aventis, Frankfurt am Main, Germany). Post-stimulation samples were collected 30, 45, 60, and 90 min after the injection to measure luteinizing hormone (LH), follicle stimulating hormone (FSH), and estradiol (E2) levels. Serum LH and FSH levels were measured using immunoradiometric assay (BioSource, Nivelles, Belgium). The LH and FSH assay detection limits, intra-assay coefficients of variation (CVs), and inter-assay CVs were 0.1 and 0.2 IU/L, 1.4–3.9% and 1.1–2.0%, and 3.4–8.0% and 2.4–4.4%, respectively. E2 levels were determined on radioimmunoassay with analytical sensitivity of 5 pg/mL, and the limit of detection of E2 was 20 pg/mL (RIA; Coat-A-Count, Diagnostic Products, Los Angeles, CA, USA).

All of the patients received a 3.75 mg leuprolide acetate depot subcutaneously every 28 days for at least 1 year. The patient’s pubertal status (Tanner stage for breast development) was assessed and documented by one pediatric endocrinologist. Patients were categorized according to their pubertal stage (Tanner II-V). The bone age was measured using the method described by Greulich and Pyle [8]. The body mass index (BMI) was calculated, and the standard deviation scores (SDSs) of the anthropometric measurements were determined using the 2017 Korean National Growth Charts [9].

This retrospective study was approved by the institutional review board of Ajou University Hospital (AJIRB-MED-OBS-15-250). The need for informed consent was waived due to the retrospective design, and all of the measurements were performed as part of routine practice. The study adhered to the tenets of the Declaration of Helsinki.

### 2.4. Statistical Analysis

Statistical analyses were performed using IBM SPSS Statistics version 25.0 (IBM Corp., Armonk, NY, USA). An independent t-test was used to compare the differences between the patient and control groups. There was a significant correlation between the SEs for the right and left eyes in the Pearson’s correlation analysis (*r* = 0.955, *p* < 0.001). Thus, this study used the data from the right eye. For the patients with CPP, univariate regression analysis between the ophthalmic and anthropometric measurements was performed. In addition, multiple linear regression was performed with stepwise variable selection, including the age at first evaluation, change in weight and height over one year, peak LH, FSH, and E2 levels to detect significant associations with the changes in the ALs and SEs during the GnRHa treatment. A *p*-value < 0.05 was considered statistically significant.

## 3. Results

A total of 32 subjects with a mean age of 8.6 ± 0.7 years (range, 6.7–10.2 years) were assessed over a 1-year period. Table 1 gives the anthropometric measurements of 18 patients with CPP and 14 age-matched normal controls. The weight and BMI SDSs did not differ between the two groups. However, the height SDS and bone age were significantly higher in patients with CPP compared with the controls. Out of 18 patients with CPP, the majority of subjects were at Tanner stage 2 (*n* = 16, 88.8%), and 2 (11.2%) children were at at Tanner stage 3. All of the control subjects were prepubertal (Tanner stage 1). The mean peak LH, FSH, and E2 levels in the CPP patients were 12.0 ± 7.2 IU/L, 12.4 ± 3.7 IU/L, and 57.4 ± 11.9 pg/mL, respectively.

Table 2 shows the myopic progression of both the CPP patients and controls. At base line, there was no significant difference in the SEs and ALs between the two groups. After 1-year, at the mean age of 9.6 ± 0.9 years, both the patients with CPP and controls showed myopic progression, with an average myopic shift of −0.73 ± 0.48 D, and AL elongation with a mean change of 0.44 ± 0.61 mm. The annual changes in the SEs and ALs were greater in the controls than those in the patients with CPP, which was not statistically significant different. 

For the patients with CPP, the annual change in AL correlated significantly with the changes in height and the difference between bone age and chronological age (BA-CA) in the univariate analysis (Table 3). Moreover, multivariate linear regression analysis showed that the annual AL change was significantly correlated with the change in height (β = 0.691, *p* = 0.039). The annual change in the SEs did not correlate significantly with any puberty parameters, including the height, weight, and BMI; bone age; and pubertal hormones (data not shown).

## 4. Discussion

In this study, the patients with CPP who were at the risk of myopic progression showed a similar myopic progression after receiving GnRHa treatment for 1 year compared with the normal controls. In addition, we found that there was a trend of less myopic progression in the CPP patients with treatments than controls, and for the CPP patients, the annual change in ALs was positively correlated with the annual change in the height. 

Once myopia develops, myopia may continue to progress throughout childhood and adolescence [2]. Particularly, the prevalence of myopia and rate of myopic progression were higher in girls than in boys. Moreover, myopic progression was faster in girls with earlier onset puberty than those with later onset puberty [10]. There are several hypotheses regarding the rapid progression of myopia in girls during puberty. Elevation in the pubertal hormones such as estrogen may affect myopic progression given that the presence of sex hormone receptors in human eye tissues [11]. Wang et al. [12] reported that estradiol levels were significantly correlated with the axial length elongation and myopic shift. Another possible explanation is that the increase in the height velocity during puberty is associated with myopia progression. In our study, annual axial elongation was positively correlated with annual height gain in the CPP patients after adjusting for age; weight; and pubertal hormones such as LH, FSH, and E2. A study by Wang et al. [13] reported that longitudinal changes in AL and height occurred concomitantly in Chinese children. In a study of 1779 school children (aged ages 6 to 14 years), Yip et al. [3] assessed the association between the mean age at the peak height and the AL or SE velocity. They reported that children with earlier growth spurts experienced earlier peak SE and AL velocities compared to those with later onset growth spurts. Recently, a negative correlation between height and subfoveal choroidal thickness in girls aged 11 to 12 years was also reported [14]. These studies supported the hypothesis that growth spurts during puberty in girls are at higher risk for faster myopia progression. Therefore, GnRHa treatment, which can suppress pubertal hormones and growth spurts, may influence the myopic progression of girls with CPP. In our study, the annual changes in AL and SE were not significantly different between girls with CPP who were treated with GnRHas and the controls, while the CPP patients with treatment tended to have less myopic progression than controls. For ethical reasons, we only included the CPP patients who underwent treatments, so the myopic progression in the CPP patients without treatments was not able to be shown. However, based on the studies for myopia controls, the myopia in children with school ages progressed annually in −0.81 D or −0.60 D, which was greater than or similar to −0.64 ± 41 D of our CPP patients [15,16]. Therefore, we suggested the possibility that GnRHa treatment may retard myopic progression in the CPP patients. Long-term follow up and large cohort studies are required to validate the association between myopic progression and GnRHa treatment. 

Several limitations stem from the retrospective case-control study design used here. First, we did not evaluate the known variables related to myopic progression such as the time spent participating in outdoor activities, parental myopia history, the children’s education, and near work load. Additionally, we did not evaluate estrogen levels after 1 year of GnRHa treatment. Therefore, it was difficult to determine causality. Second, the sample size was small and the follow-up period was relatively short. Despite these limitations, to the best of our knowledge, this is the first study to analyze myopic progression in patients with CPP who were receiving GnRHa treatment.

In conclusion, the changes in the ALs and SEs in girls with CPP who were receiving GnRHa treatment were similar with the normal controls. Since puberty is an important period for myopia progression, a regular ophthalmic evaluation may be necessary particularly in patients with CPP. In addition, we suggest GnRHa treatment may be beneficial in slowing down myopic progression in girls with CPP. 

## Figures and Tables

**Table 1:** Anthropometric measurements at baseline and after 1 year.

	Precocious Puberty (*n* = 18)	Control (*n* = 14)	*p* Value
At Baseline			
Age (years)	8.7 ± 0.5	8.3 ± 0.9	0.108
Height SDS	0.72 ± 0.68	0.15 ± 0.72	<0.031
Weight SDS	0.63 ± 0.75	0.34 ± 1.15	0.384
BMI SDS	0.40 ± 0.91	0.38 ± 1.26	0.943
Tanner stage			
I	0	14	
II	16	0	
III	2	0	
Bone age (years)	10.4 ± 0.9	9.1 ± 1.0	0.002
BA-CA (years)	1.6 ± 0.7	0.8 ± 0.7	0.006
At 1-Year Follow-Up			
Age (years)	9.8 ± 0.4	9.3 ± 1.2	0.263
Height SDS	0.88 ± 1.70	0.11 ± 0.83	0.132
Weight SDS	0.75 ± 1.14	0.19 ± 1.03	0.165
BMI SDS	0.55 ± 1.00	0.21 ± 1.01	0.339
Bone age (years)	11.2 ± 0.6	10.2 ± 0.9	0.001
BA-CA (years)	1.6 ± 1.2	0.8 ± 0.8	0.049

SDS, standard deviation score; BMI, body mass index; BA-CA, the difference between bone age and chronological age.

**Table 2:** Myopic progression over 1 year.

	Precocious Puberty (*n* = 18)	Control (*n* = 14)	*p* Value
At baseline			
Spherical equivalent (D)	−1.43 ± 1.40	−1.12 ± 1.44	0.542
Axial length (mm)	23.96 ± 1.12	23.52 ± 1.14	0.280
At 1-year follow-up			
Spherical equivalent (D)	−2.08 ± 1.52	−1.97 ± 1.52	0.842
Axial length (mm)	24.32 ± 1.13	24.07 ± 1.12	0.544
Annual change of SE (D)	−0.64 ± 0.41	−0.85 ± 0.55	0.246
Annual change of AL (mm)	0.35 ± 0.22	0.55 ± 0.89	0.373

D, diopters; SE, spherical equivalent; AL, axial length.

**Table 3:** Univariate and multivariate analysis of factors associated with annual change of axial length in girls treated with gonadotropin-releasing hormone agonist (*n* = 18, *r*^2^ = 0.478, *p =* 0.039).

Parameter	Univariate		Multivariate	
	*r*	*p* Value	*β*	*p* Value
Annual change of height	0.511	0.030	0.691	0.039
Annual change of weight	0.423	0.080		
Annual change of BMI	0.277	0.266		
Age at baseline	−0.088	0.729		
Peak LH	−0.060	0.813		
Peak FSH	0.010	0.969		
Peak E2	−0.508	0.162		

SDS, standard deviation score; BMI, body mass index; LH, luteinizing hormone; FSH, follicle stimulating hormone; E2, estradiol. Stepwise multivariate regression analysis contained the following independent variables entered into the model: age at first evaluation, the change in weight and height for a year, peak LH, peak FSH, and peak E2.

## Data Availability

All relevant data are included in the paper and its Supporting Information files. Otherwise, the raw data analyzed during the current study are not publicly available as owners gave their written consent only to use the data for the current study on IRB approve. The datasets used and/or analyzed in the present study are available from the corresponding author on reasonable request.
